# Mesalazine-associated lung fibrosis: case report and literature review

**DOI:** 10.1016/j.rmcr.2025.102284

**Published:** 2025-09-16

**Authors:** Nazlı Zeynep Uslu, Mustafa Hasan Adleh, Ebru Bilir, Fahad al-deen Kata, Merih Kalamanoğlu Balcı

**Affiliations:** a Özel Medical Park Göztepe Hastanesi, Istanbul, Turkey; b Department of Medicine, Bahçeşehir Üniversitesi, Istanbul, Turkey

**Keywords:** Lung fibrosis, Mesalazine adverse effects, 5-ASA lung fibrosis

## Abstract

**Background:**

Even though uncommon, drug-induced interstitial lung disease (DIILD) represents a serious adverse drug reaction. We report a case of a patient with a history of ulcerative colitis who was receiving mesalazine.

**Presentation:**

The patient developed dyspnea, hypoxemia, and respiratory failure accompanied by resting oxygen desaturation. Initial CT imaging revealed bilateral perihilar ground-glass opacities along with focal areas of consolidation. Inflammatory markers were elevated, but procalcitonin levels remained persistently low; sputum cultures and multiplex PCR ruled against an infectious origin. Mesalazine was discontinued, and the patient's respiratory status improved dramatically. Follow-up CT revealed interval resolution of the pulmonary lesions, and a diagnosis of DIILD was clinically established.

**Conclusion:**

This case emphasizes the importance of balancing clinical suspicion between steroid-related infectious risk and an uncommon drug reaction. We also identified similar Mesalazine-associated DIILD cases in the literature to demonstrate similar radiologic patterns, onset time, and outcomes.

## 1 Introduction

In earlier times, sulfasalazine (sulfapyridine and 5-ASA) was the treatment of choice for ulcerative colitis (UC) patients (with or without the addition of corticosteroids depending on the individual clinical condition of the patients), however, due to the high prevalence of adverse reactions associated with sulfasalazine, it was widely disregarded, especially with the introduction of mesalazine (5-ASA) which had fewer adverse events [1]. Even though the adverse effects attributed to mesalazine are milder in nature and fewer than those of sulfasalazine, there have been some rare but serious adverse effects reported in the literature.

## 2 Case presentation

### 2.1 Patient history

A 72-year-old female, former smoker, was diagnosed with ulcerative colitis (UC) presenting as mild to moderate pancolitis on February 17. She was discharged on a regimen of mesalazine 1 g twice daily and 40 mg prednisolone tablet daily. Her condition was initially well controlled under this treatment, and no adverse drug reactions were reported.

### 2.2 Presentation and findings

**Fig. 1:**
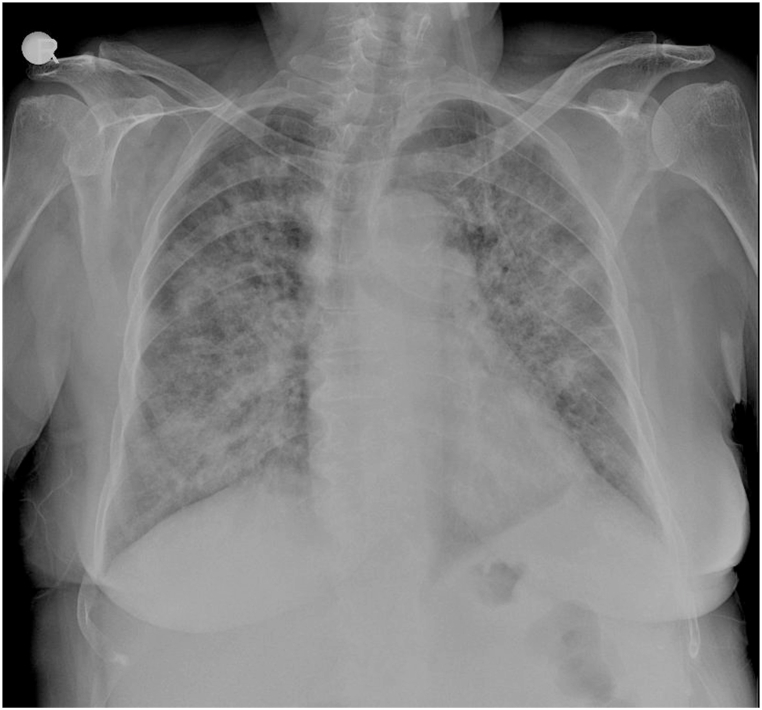
An X-ray film shows ground-glass opacities bilaterally.

A 72-year-old female with a diagnosis of UC presented to the emergency department at a private hospital in Istanbul on the 10th of April with severe fatigue and was found to have impaired consciousness. She had an intermittent, persistent cough, dyspnea, and hypoxia, and was in a state of respiratory insufficiency, with an oxygen saturation (SpO2) of 84 % at rest; she also had tachypnea (worsening O2 needs). Upon physical examination, the patient was noted to have generalized weakness, and auscultation revealed bilateral diffuse rales. The patient was subsequently admitted to the hospital and initiated on supplemental oxygen therapy via nasal cannula at a flow rate of 4 L/min for respiratory support. Initial X-ray findings showed bilateral generalized ground-glass opacities [Fig. 1].On the first hospital day, prednisolone was decreased to 8 mg, ciprofloxacin 400 mg IV twice daily and ceftriaxone 1 g IV twice daily (subsequently discontinued on the 3rd hospital day) were started for a presumed pneumonia infection.

Over the next 2 days, the patient was followed up for respiratory symptoms, with a persistent need for oxygen support. Antibiotics and steroid therapy continued as before until the third day.

On the 4th day, the patient's respiratory symptoms were gradually worsening, and required additional oxygen support to maintain adequate oxygenation; hence, the patient was switched to a high-flow nasal cannula (HFNC) on FiO2 50 % to maintain SpO2 between 92 % and 94 %.

Additionally, the gastroenterology department was consulted about possible medication adverse reaction. It was then decided that mesalazine should be discontinued while observing the patient for any possible improvement. In the meantime, the patient commenced methylprednisolone 40 mg IV twice daily, while continuing ciprofloxacin 400 mg IV twice daily. Piperacillin-tazobactam 4.5 g IV every 8 hours and sulfamethoxazole/trimethoprim (TMP-SMX) 1200/240 mg IV every 6 hours were added empirically to have a broader antibacterial coverage.

Over the next 4 days, the patient's condition dramatically improved, with marked improvement in oxygenation status, and the patient restored her ability to freely ambulate as fatigue had almost resolved.

### 2.3 Investigations

Blood work-up was almost inconclusive and displayed minimal signs of infection with mild neutrophilia. However, since the day of admission, it was noted that CRP (C-reactive protein) levels were gradually increasing throughout the patient's stay in the hospital, till the day mesalazine was discontinued. CRP reached its highest value on the 3rd day of hospitalization at 220 mg/L, and on the 4th day (when mesalazine was discontinued), it witnessed a gradual, sudden decline that continued till discharge day, when it returned to its normal value. On the other hand, serial procalcitonin testing yielded repeated low levels (0.06–0.10 ng/mL). Sputum cultures returned negative test results, and a multiplex PCR that included 22 pathogenic organisms was also negative. Thus, infectious causes were effectively ruled out. However, since the patient's condition necessitated continuous oxygen support, bronchoscopy could not be carried out.

**Fig. 2:**
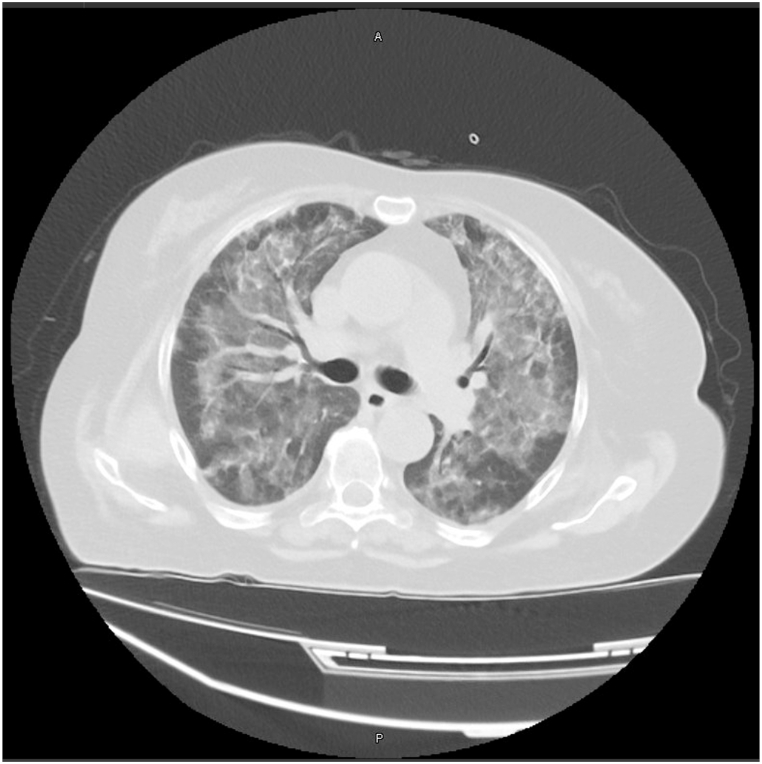
Axial CT scan taken before Mesalazine was discontinued, demonstrating diffuse consolidation of the lungs with ground-glass lesions.

**Fig. 3:**
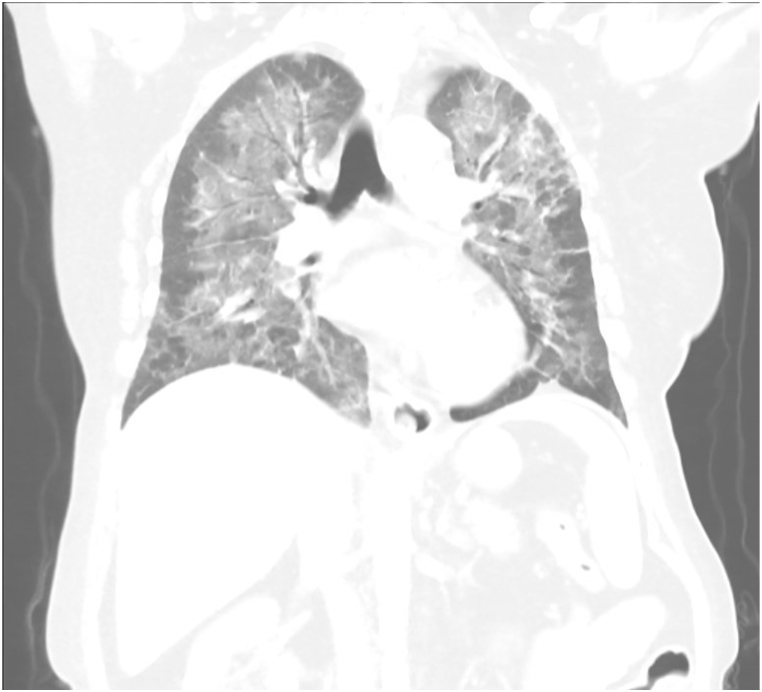
Coronal CT scan showing similar findings as seen in Fig. 2.

Aside from the X-ray that was acquired on admission, the patient also had a high-resolution computed tomography (HRCT) on the third day of hospitalization that revealed bilateral-perihilar ground-glass opacities, and widespread areas of consolidation, with ground-glass findings that correspond to pulmonary fibrosis. The findings of the HRCT seemed to be in a worse state when compared to the initial X-ray, indicating the rapid deterioration that occurred throughout the patient's hospitalization [Fig. 2], [Fig. 3].

**Fig. 4:**
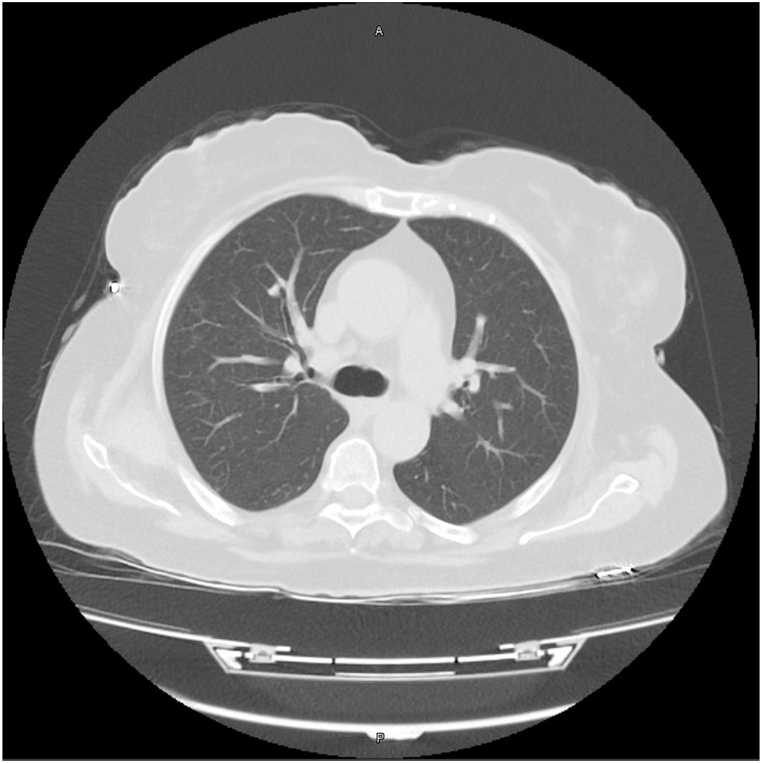
A follow-up Axial CT scan after a month of Mesalazine discontinuation showing complete resolution of the previously described lung lesions.

**Fig. 5:**
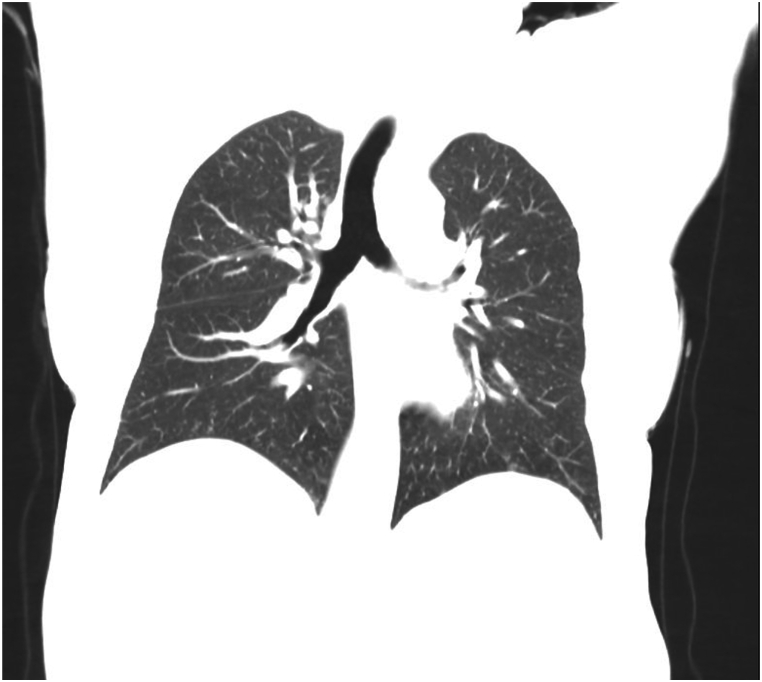
Coronal CT scan further demonstrating the recovery of the lung parenchyma.

Another HRCT that was taken 3 days after the discontinuation of mesalazine showed almost total resolution of pulmonary fibrosis, with marked absence of areas of consolidation. One month later, a follow-up CT demonstrated total resolution of the pulmonary lesions and showed normal lung parenchyma [Fig. 4], [Fig. 5].

### 2.4 Follow-up

The patient was discharged from the hospital on the 9th day after her condition stabilized; both lab tests and radiological findings displayed total resolution of the previous abnormalities, mesalazine was decided to be avoided in the management of her UC condition.

### 2.5 Steroid & risk of infection

The dose-response association between corticosteroid use and the increased risk of infections or Pneumocystis *jirovecii* pneumonia (PJP) is a widely recognized phenomenon [2,3]. Therefore, our patient was empirically started on broad-spectrum antibiotics (piperacillin-tazobactam) and Trimethoprim-Sulfamethoxazole (TMP-SMX). Ciprofloxacin was continued to cover any possible Pseudomonas infection. Until the fourth hospital day, prednisone was tapered to 8 mg. Once diagnostic tests had ruled out the possibility of infection, high-dose methylprednisolone therapy was initiated.

### 2.6 Literature review

We have identified some cases in the literature that are relevant to our presented case and could draw out a resemblance in both the pattern of lung injury associated with the use of mesalazine, and the presentation of the patient.

Alskaf et al. (2013) reported a case of 82-year-old man with a diagnosis of Crohn's disease, who reported to the emergency department 9 days after he was started on a treatment regimen that included mesalazine. High-resolution CT of the patient showed evidence of lung fibrosis. After ruling out possible infectious causes, the treatment was discontinued and the patient's clinical picture improved greatly within days, with a later complete resolution of the pulmonary fibrosis [4].

Kotsiou and Gourgoulianis (2019) [5] also published a paper describing a 55-year-old male who started having respiratory symptoms that eventually resulted in hypoxemic respiratory failure. The patient was on mesalazine for 4 months, and glucocorticoids for 3 months. The high-resolution CT of the patient revealed diffuse micronodular pattern, with infectious sources ruled out, the treatment was discontinued, and the patient experienced an accelerated recovery, with radiological imaging revealing reversal of the previously described lesions.

Lázaro and García-Tejero (1997) [6] described one of the earliest cases of lung injury attributed to the use of mesalazine. They reported a 60-year-old male with a diagnosis of UC, who was started on the drug 4 weeks before complaining of dyspnea, cough, and fever. Infectious aetiologies were excluded, and the patient's X-ray showed bilateral interstitial infiltrates, also the patient has reduced DLCO (diffusing capacity for CO). After stopping the therapy, the patient's condition rapidly improved, and subsequent radiology imaging revealed the resolution of the abnormalities noticed before.

However, Moeser et al. (2015) described in their article that the typical lung involvement pattern in the cases of mesalazine-induced lung toxicity had eosinophilia as a supportive finding [1]. Furthermore, as a part of the cases they reported to having respiratory side-effects attributed to the drug's use, they described 14 cases that had eosinophilia identified in the serological tests, and/or eosinophilic infiltrates on lung biopsy and in BAL (bronchoalveolar lavage) test, suggesting a strong link between the presence of eosinophilia, and the likelihood of DIILD. On the other hand, our case had no signs of eosinophilia.

Even though lung involvement as an adverse event of mesalazine is rare, these 3 cases reveal a clear resemblance in the pattern of lung involvement (both clinically and radiologically) in patients receiving the treatment, with very similar style of recovery following the discontinuation of the drug. Our patient also experienced a comparable clinical course with these reported cases, raising the suspicion that the 5-ASA treatment might have an underrecognized pulmonary side effect.

## 3 Discussion

Albeit mesalazine's adverse reactions are much less frequent than those observed with the use of sulfasalazine, patients on mesalazine should still be monitored for possible adverse effects. In a study comparing the frequency of adverse events reported by patients who are on sulfasalazine to those on mesalazine, it was found that 29 % of the patients who were prescribed sulfasalazine reported medication adverse reactions, compared to only 15 % of the patients in the mesalazine group [7]. Also, the same study concluded that treatment withdrawal was much higher in the sulfasalazine group when compared to the mesalazine group (13 % and 5 % respectively) [7].

The pathophysiology of lung injury resulting from mesalazine use is still unknown, with some authors suggesting it is a hypersensitivity reaction (dose-independent), others claim that it is an adverse drug reaction and is dose-dependent [8].

Given the close temporality between starting mesalazine and the emergence of respiratory complaints, paired with the new radiological findings of the patient during this period and the negative infectious work-up (PCR and sputum cultures); which was followed by a rapid improvement after stopping mesalazine, our patient was diagnosed with drug-induced interstitial lung disease (DIILD) clinically without the need for further confirmatory testing. A similar approach was described prior in the literature, that also points to the lack of defined guidelines for management of DIILD [9].

Bronchoalveolar lavage (BAL) was deferred in our case due to the patient's continuous need for oxygen support.

Concerning the previously mentioned cases in the literature review section, it should be noted that in our case and both cases of Lázaro and García-Tejero (1997) [6], the interval between starting mesalazine therapy and the emergence of pulmonary adverse reactions was 2 months, however, in the case of Kotsiou and Gourgoulianis (2019) [5], pulmonary symptoms were only reported after 4 months since commencing mesalazine. However, the same pattern of rapid patient recovery after discontinuing the medication was very similar in all 3 cases as well as ours (days to a month).

On the other hand, both the cases of Kotsiou and Gourgoulianis (2019) [5] and Lázaro and García-Tejero (1997) [6] reported eosinophilia accompanying the clinical findings in their cases. However, our patient's lab reports revealed no signs on eosinophilia.

It should also be mentioned that the difference between doses and the development of adverse events was irrelevant in the reviewed cases. Even though in the case of Kotsiou and Gourgoulianis (2019) [5], the patient was on 4 g of mesalazine daily, but developed respiratory symptoms only 4 months later, contrary to our patient, who was receiving 2 g of mesalazine but developed symptoms 2 months after the therapy was started. Also, according to Alskaf et al. (2013) [4], their patient was receiving a daily dose of 3 g of mesalazine but developed symptoms 9 days after commencing therapy. Therefore, this should raise concerns whether a dose-dependent relationship truly exists in the cases of lung toxicity related to mesalazine (5-ASA) use, and whether it could be attributed to the severity of the symptoms and findings in such situations.

We concluded that further research is needed to better understand the pathophysiology and the nature of these adverse effects in relation to mesalazine use, and how they can be avoided in the future. We also suggest that patients who are on a mesalazine regimen should be followed up periodically for lung toxicity, or any additional adverse events of the drug.

## CRediT authorship contribution statement

**Nazlı Zeynep Uslu:** Writing – review & editing, Writing – original draft, Resources, Project administration, Investigation, Formal analysis, Data curation, Conceptualization. **Mustafa Hasan Adleh:** Writing – review & editing, Writing – original draft, Validation, Methodology, Investigation, Data curation, Conceptualization. **Ebru Bilir:** Writing – review & editing, Writing – original draft, Methodology, Investigation, Data curation, Conceptualization. **Fahad al-deen Kata:** Writing – review & editing, Writing – original draft, Validation, Investigation, Data curation, Conceptualization. **Merih Kalamanoğlu Balcı:** Supervision, Formal analysis, Data curation, Conceptualization.

## Informed consent

Written informed consent was obtained from the patient for their anonymized information to be published in this article.

## Ethics approval

in accordance with the regulation of ministry of health in Turkey, our institution does not require ethical approval for reporting individual cases or case series (observational studies), as stated by the ethics committee of our institution; Bahçeşehir University Clinical Research Ethics Committee.

## Data availability statement

The corresponding author may provide the data supporting this study upon request because it was acquired from the private hospital's records.

Permission to reproduce material from other sources: no materials were acquired from other sources.

## Clinical trial registration

Not applicable in the scope of our research.

## Funding sources

No specific grant from funding agencies was received for this work.

## Declaration of competing interest

The authors declare that they have no known competing financial interests or personal relationships that could have appeared to influence the work reported in this paper.
